# RIPK1 in the inflammatory response and sepsis: Recent advances, drug discovery and beyond

**DOI:** 10.3389/fimmu.2023.1114103

**Published:** 2023-04-05

**Authors:** Xiaoyu Liu, A-Ling Tang, Jie Chen, Nan Gao, Guoqiang Zhang, Cheng Xiao

**Affiliations:** ^1^ Department of Emergency, China-Japan Friendship Hospital, Beijing, China; ^2^ China-Japan Friendship Hospital, Chinese Academy of Medical Sciences and Peking Union Medical College, Beijing, China; ^3^ Graduate School, Beijing University of Chinese Medicine, Beijing, China

**Keywords:** RIPK1, sepsis, cytokine storm, inflammatory response, PANoptosis

## Abstract

Cytokine storms are an important mechanism of sepsis. TNF-α is an important cytokine. As a regulator of TNF superfamily receptors, RIPK1 not only serves as the basis of the scaffold structure in complex I to promote the activation of the NF-κB and MAPK pathways but also represents an important protein in complex II to promote programmed cell death. Ubiquitination of RIPK1 is an important regulatory function that determines the activation of cellular inflammatory pathways or the activation of death pathways. In this paper, we introduce the regulation of RIPK1, RIPK1 PANoptosome’s role in Inflammatory and sepsis, and perspectives.

## 1 Introduction

Sepsis is a life-threatening condition characterized by organ dysfunction resulting from a dysregulated host response to infection. Cytokine storm, caused by imbalances in cytokines such as IL-1β, IL-6, and TNF-α, disrupts the normal immune balance, ultimately leading to organ dysfunction. TNF-α, a well-studied cytokine, is secreted by macrophages within 30 minutes after infection (1) and activates inflammatory pathways such as NF-κB, MAPK, and JNK signaling, or programmed cell death (2). RIPK1, a nodal protein in the TNF pathway, binds to TNFR1 at its intracellular terminus after TNF-α binds to TNFR1, subsequently recruiting TRAF1, cIAP1/2, LUBIC, and other E3 ubiquitinates to form proinflammatory complex I, thereby activating inflammation-related pathways. Deubiquitination of RIPK1 by CYLD, OTULIN, and A20 leads to the phosphorylation of RIPK1 and formation of death complex II, which mediates programmed cell death (3). RIPK1 has been implicated in TNF-induced cell death and proposed as a target for cancer therapy to induce cancer cell death (4, 5). Recent studies have elucidated the important role of TNF-mediated necroptosis in the inflammatory response, and RIPK1 inhibition has shown therapeutic effects on various rheumatic immune diseases as well as infections. However, the mechanism regulating RIPK1 in sepsis remains unclear. This manuscript centers on the impact of RIPK1 regulation induced by sepsis on the inflammatory response, and the current state of medical research.

## 2 Structure and function of RIPK1

**Figure 1:**
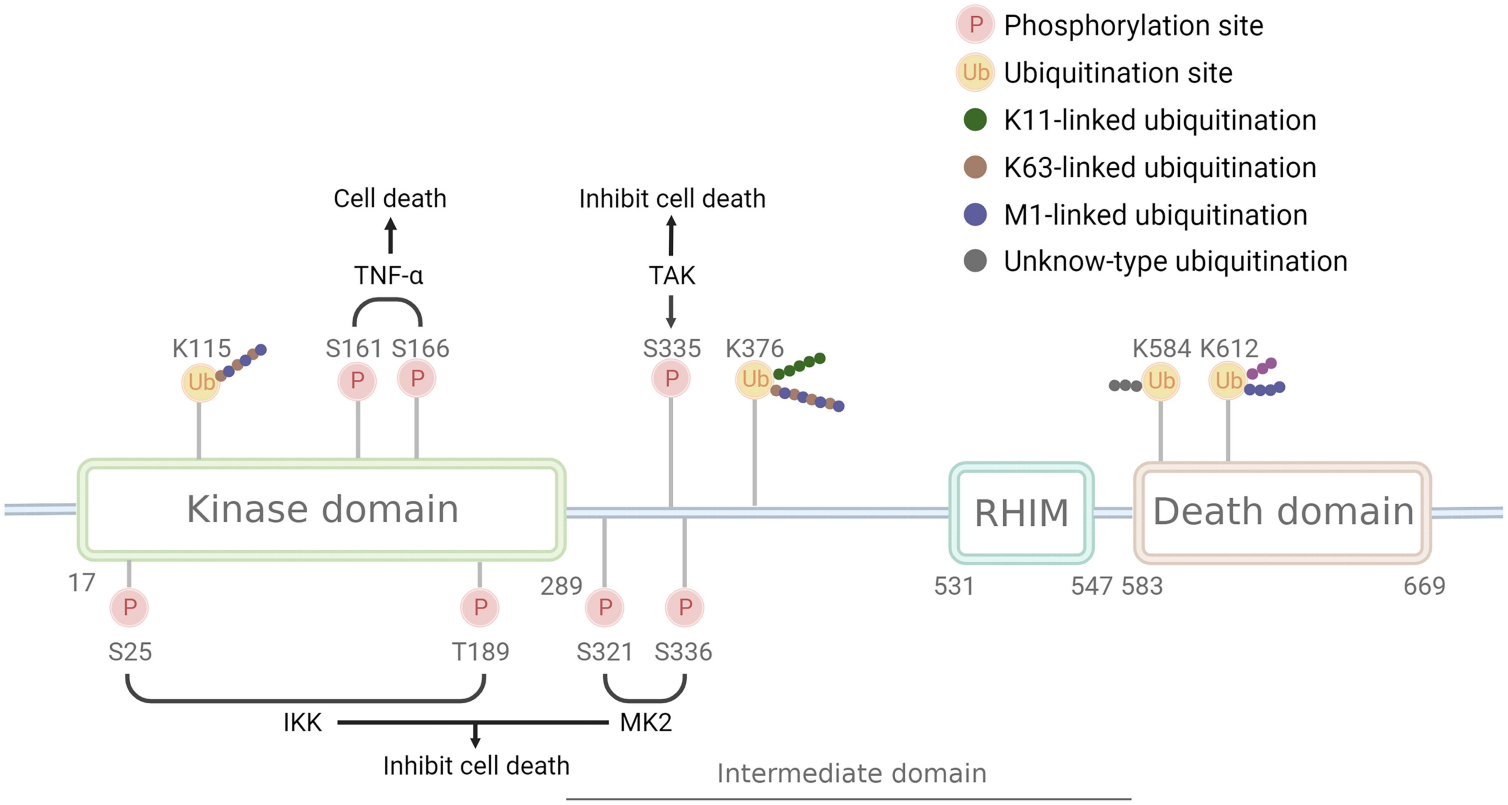
Structure and modification sites of RIPK1. RIPK1 comprises the N-terminal kinase domain, the C-terminal death domain that mediates death signaling, the bridging intermediate domain, and the RIP homotypic interaction motif (RHIM). Phosphorylation sites have two opposite effects: S161 and S166 have a pro-death effect and are phosphorylated after TNF-αstimulate, while S25, T189, S321, S335, and S336 have a pro-survival effect and are phosphorylated by IKK, MK2, and TAK. Ubiquitination sites can be linked by different Ub chains: K115 can be linked by K63 and M1 chain, K376 linked by K11, K63, and M1 chain, K612 linked by M1 chain, while K584 remains unknown. Pink circle with P indicates phosphorylation sites, a yellow circle with Ub indicates ubiquitination sites, green dots indicate K11 ub chain, brown dots indicates K63 ub chain, purple dots indicate M1 ub chain, grey indicates unknown ub chain.

RIPK1, the first member of the receptor-interacting Ser/Thr kinase (RIPK) family, is a crucial protein involved in the cell death process (1, 2). Its structure (**Figure 1**) comprises the N-terminal kinase domain, the C-terminal death domain that mediates death signaling, the bridging intermediate domain, and the RIP homotypic interaction motif (RHIM) (3–5). RIPK1 binds to the DD domains of TRADD and TNFRSF to form a trimeric structure, and TRADD then recruits E3 ubiquitin ligases such as cIAP1/2 and LUBAC to form a complex I scaffold structure, exerting proinflammatory effects (6). When the deubiquitinating enzymes A20, CYLD, and OTULIN degrade the ubiquitin chain of RIPK1, complex I dissociate, and the released RIPK1 forms the cytoplasmic death-induced signaling complex (DISC), Complex IIa, or complex IIb, inducing programmed cell death (7). RIPK1 has complex functions in cells, including the regulation of cell death associated with its kinase activity and regulation of pro-survival inflammatory pathways associated with its scaffold structure independent of its kinase activity. RIPK1 is constitutively located in the cytoplasm of cells (8). However, nuclear translocation of RIPK1 has also been reported (9–12), although its function in the nucleus has not been clarified.

In recent years, the inflammatory response and necroptosis induced by RIPK1 have been extensively studied and are involved in the pathogenesis of various autoimmune and chronic inflammatory diseases, such as neurodegeneration, ischemia-reperfusion injury, severe infections, cardiovascular disease, chronic obstructive pulmonary disease, skin inflammation, systemic lupus erythematosus, inflammatory bowel disease, rheumatoid arthritis, and psoriasis (13–22). Meanwhile, inhibiting RIPK1 by necrostatin-1 reduced TNF-α-affected model in mouse mortality, cell death, and persistent inflammatory responses (23). But interestedly, Patients with biallelic inactivating mutations in RIPK1, including missense, nonsense, and frameshift mutations, exhibit symptoms of inflammatory bowel disease and combined immunodeficiency, develop lymphopenia, and display increased sensitivity to TNFR1-mediated cell death (21, 22). RIPK1 knockout mice are embryonic lethal and exhibit severe *in vivo* inflammatory responses that activate apoptosis and necroptosis pathways (24). In summary, RIPK1 both triggers cytokine release and cell death. However, the underlying mechanism by which RIPK1 leads to changes in cytokine release remains unclear.

## 3 Regulation of RIPK1

RIPK1 is subject to regulation by a variety of enzymes and modifications, including ubiquitination and phosphorylation (**Table 1**). Ubiquitination can activate multiple inflammatory pathways and inhibits kinase activity, thereby reducing cell death. The kinase activity of RIPK1 is responsible for inducing programmed cell death (25). Mutations at the K45A site, which result in a loss of kinase activity, protect cells from TNF-α-stimulated necroptosis (26). Similarly, the D138N mutation, which also causes a loss of kinase activity, confers resistance to TNF-α induced necroptosis (27), and rats carrying this mutation are resistant to TNF-α-induced shock (28), however, Newton et.al (29). found RIPK1 D138N catalytically inactive mice were as sensitive to LPS challenge as wildtype mice, but were resistant to body temperature loss after TNF challenge. Numerous studies have identified S161 and S166 as important phosphorylation sites in RIPK1, and the crystal structure of RIPK1 indicates that S161 is located in the T-loop structure in an open conformation, suggesting that phosphorylation at this site promotes interaction between RIPK1 and RIPK3 and initiates the occurrence of necroptosis (30). Autophosphorylation at S166 induces TNF-mediated inflammatory responses in a variety of animal models (31). Mutations at the aforementioned sites (K45, S166, S161, and D138) all reside in the kinase domain of RIPK1, underscoring the critical role of RIPK1 kinase activity in necroptosis, and these mutations promote the systemic inflammatory response both *in vivo* and *in vitro*. Therefore, RIPK1 kinase activity may contribute to TNF-α-induced necroptosis and exacerbation of the inflammatory response.

**Table 1:** Ubiquitin and phosphoryl sites of RIPK1.

Sites	Modification type	Associated enzyme	Action	Ref.
S25	Phosphorylation	Phosphorylation: IKK Dephosphorylation: PPP1R3G	Inhibition of kinase activity and necroptosis	(32, 33)
S161, S166	Phosphorylation	Autophosphorylation	Induction of necroptosis and inflammatory response	(30, 31)
T189	Phosphorylation	Phosphorylation: IKK	Inhibition of kinase activity	(34)
S321, S336 (Human S336)	Phosphorylation	Phosphorylation: MK2, TAK	Inhibition of kinase activity	(35–37)
K45, D138	K45A mutation; D138N mutation	–	Kinase active site, promotes TNF-α-induced cell death	(26, 29, 65)
K115	M1, K63	PELI1: K63, M1	Activates the NF-κB pathway, regulates RIPK1 autophosphorylation, RIPK3 phosphorylation, and MLKL phosphorylation	(13, 38)
K376 (Human K377)	K63, K11, M1	CIAP1/2: K63, M1 MIB2: K11, K48, K63 Parkin: K63	Activation of IKK, NF-κB, and MAPK pathways, inhibition of RIPK1 kinase activity, inhibition of complex II assembly	(13, 38, 40, 66)
K612 (Human K627)	K63, M1	LUBAC: M1	Activates NF-κB, assists RIPK1 autophosphorylation, and inhibits necroptosis in the TLR3/4 pathway	(42, 43)
K584 (Human K599)	–	–	Facilitates complex II formation and RIPK1 dimer formation	(41)

In addition, RIPK1 also contains inhibitory phosphorylation sites. Phosphorylation of the S25 site inhibits RIPK1 kinase activity, IKKs phosphorylate the S25 site in complex I and protect cells deficient in the ubiquitinating enzyme SHARPIN from death (32), and dephosphorylation of the S25 site by PPP1R3G promotes the kinase activity of RIPK1, inducing necroptosis (33). T189 also inhibits RIPK1 kinase activity through phosphorylation by IKKs (34), while the S321 and S336 (human S335) sites are phosphorylated by MK2 and TAK to inhibit its kinase activity (35–37), and phosphorylation of the aforementioned inhibitory sites requires the activation of the NF-kB or MAPK pathway. This may be a negative feedback regulation mechanism of RIPK1-associated inflammatory factor transcription.

RIPK1 possesses multiple ubiquitination sites, with K11, K48, K63, and M1 ubiquitin chains being identified in this protein. K63 ubiquitin chain of RIPK1 is crucial for complex I formation and activation of inflammatory factor transcription, such as NF-κB signaling, whereas deubiquitinated RIPK1 leads to the formation of complex II and cell death. K115 is located in the kinase domain of RIPK1, compared with the wild type, the K115R mutation showed a decrease in the synthesis of K63 and M1 ubiquitin chains, and K115R significantly decreased the phosphorylation of RIPK1, RIPK3, and MLKL, ultimately inhibiting the occurrence of necroptosis (38, 39). K376 (human K377) is located in the bridging domain, mutation of K376 promotes necroptosis and apoptosis, reduces the ubiquitination of K11, K63, and M1, hinders TNFR1 complex formation, and causes embryonic lethality (38). Mutation of K376 in mice significantly enhances TNF-α-stimulated cell death, alters TNF-induced activation of the NF-kB and MAPK pathways, accompanied by increased phosphorylation of RIPK1, RIPK3, and MLKL, resulting in embryonic death, which was reversed by inhibition of RIPK1 kinase activity (38). The K377 site is a critical ubiquitylation site for NF-κB activation and the inhibition of open reading frame 3 (ORF3) in humans (40). Mutation of K584 (human K599), located in the DD domain of RIPK1, impedes homodimerization of RIPK1 through the DD domain, inhibiting complex II formations (41). The K612R mutation inhibits RIPK1 phosphorylation, leading to the inhibition of caspase-3, caspase-8, and RIPK1 cleavage, reduced ubiquitination of complex I, attenuated NF-κB activation, and impaired complex IIa formation, ultimately inhibiting MLKL phosphorylation. The K612R mutation inhibited RIPK1 kinase activity to a greater extent than NF-κB activity, resulting in an intestinal inflammatory response and splenomegaly in K612R mice (42). LPS stimulation of bone marrow-derived macrophages expressing the K612R mutant showed increased necroptosis, reflecting the inhibitory effect of the K612 site on necroptosis in the TLR3/4 pathway (42). Recent studies show that K612 site mutations in mice reduce the length of ubiquitin chains attached to the M1 site, causing systemic inflammatory responses and emergency hematopoietic myelograms. This change resulted from damage-associated molecular patterns (DAMPs) induced by TNFR activation. K612R mice transplanted with WT mouse bone marrow or K612R + TNF-/- mice showed improved outcomes (43).

In conclusion, the scaffold structure of complex I that binds initiator proteins, promotes the activation of the NF-kB, MAPK, and JNK pathways, and inhibits the kinase activity of RIPK1. whereas the ubiquitination at the K115, K376 (human K377), K612 (human K627), and K584 (human K599) sites, leads to the activation of the NF-kB pathway. Differences between these sites are mainly due to different stimuli or ubiquitin chain lengths. Mutations in RIPK1 ubiquitination sites, which normally enhance the kinase activity of RIPK1, lead to the activation of programmed cell death.

## 4 Regulation of RIPK1 with Inflammatory and Rheumatic Immune Diseases

RIPK1 has been implicated in the regulation of rheumatic immune diseases and chronic inflammatory responses, with its scaffold structure and kinase activity serving as key factors. Excessive ubiquitination can lead to the overactivation of the inflammatory response (44), while excessive deubiquitination can trigger necroptosis and subsequently elicit an excessive inflammatory response (45). Thus, the normal function of RIPK1 may hinge on maintaining a delicate balance between its scaffold structure and kinase activity. Specifically, deubiquitination is necessary for RIPK1’s kinase activity to manifest, and as such, the activity of the ubiquitin ligase that regulates RIPK1 may represent a crucial factor for controlling this balance (**Table 2**).

**Table 2:** Ubiquitinases and deubiquitinases of RIPK1 and its effect.

Type of RIPK1 Regulation	Regulatory Enzyme (Gene Name)	Alteration	Effect	Ref.
Ubiquitination: K63, M1	cIAPs (BIRCs)	Increased expression	Inflammatory bowel disease, rheumatoid arthritis	(49, 50)
Ubiquitination: M1	HOIL-1 (RBCK-1)	Site mutation	Premature immunodeficiency, autoimmune disease, inability to synthesize the LUBAC complex	(52, 67)
Ubiquitination: M1	SHARPIN (SHARPIN)	Inactivating mutation	TNF-dependent multiorgan inflammation, skin inflammation	(53, 55)
Deubiquitination: K63	A20 (TNFAIP)	Loss of function in heterozygotes (HA20)	Behcet’s disease, juvenile idiopathic arthritis or rheumatoid arthritis, periodic fever with aphthous pharyngitis and adenitis, autoimmune thyroiditis, Crohn’s disease, SLE, and adult Stills disease with cardiovascular symptoms, nephrotic syndrome, vasculitis, respiratory tract infection	(60, 61)
Deubiquitination: K63, M1	CYLD (CYLD)	Gene mutation	Brooke-Spiegler syndrome, familial cylindromatosis, and multiple familial trichoepitheliomas	(62)
Deubiquitination: M1	OTULIN	Functional defects	Autoinflammation, panniculitis, and dermatologic syndrome (AIPDS)	(64, 68)

cIAP1/2, an E3 ubiquitin ligase, plays a critical role in regulating NF-κB signaling and modifying the K63 and M1 ubiquitin chains of RIPK1. As a result, it regulates not only caspases and apoptosis but also numerous cellular processes, including inflammatory signaling and immunity, mitogenic kinase signaling and cell proliferation, as well as cell invasion and metastasis (46–48). Mutations in cIAP (BIRC2/3) are linked to various diseases, including tumors, autoimmunity, and inflammatory disorder (49–51). A clinical study involving individuals with inflammatory bowel disease (IBD) demonstrated nonclassical NF-κB activation with reduced responses to infliximab and adalimumab therapy, accompanied by increased expression of cIAP1/2 and various related enzymes (50). In addition, gene expression analysis revealed a significant upregulation of BIRC2 expression in individuals with rheumatoid arthritis, with cIAP1 being a central protein identified in protein-protein interaction networks linked to rheumatoid arthritis (49).

The LUBAC complex comprises HOIL-1, HOIP, and SHARPIN, with the corresponding genes being RBCK1, RNF31, and SHARPIN, respectively. This complex encodes E3 ubiquitin enzyme complexes that selectively elongate the M1 chain in RIPK1. In 2019, Oda et al. (52) identified a patient with a HOIP protein deficiency who presented with premature immunodeficiency and autoimmune inflammation. The patient carried two mutations in the RNF31 gene, and the analysis of PBMC lysates revealed that the HOIP defect resulted in secondary abnormalities in SHARPIN and HOIL-1, which prevented the formation of LUBAC complexes. Stimulation of TNF-α in PBMCs from this patient delayed activation of the NF-κB pathway, whereas IL-1β stimulation excessively activated monocytes to produce high levels of IL-6 and IL-1β. In addition, LUBAC-deficient mice exhibited enhanced sensitivity to apoptosis or necroptosis (53–56).

A20 is a deubiquitinating enzyme that targets the K63 site of RIPK1. Genome-wide association studies (GWAS) have identified single nucleotide polymorphisms (SNPs) at A20 sites that are associated with various autoimmune diseases, and it has been suggested that these SNPs may lead to reduced A20 expression (57–59). Deletion of enhancers in the A20 gene in humanized mice has been shown to increase autoantibody production, inflammation, and arthritis. Heterozygous loss-of-function mutations in A20, referred to as haploinsufficient for A20 (HA20), were first reported by Zhou et al. in 2016 (60), and subsequently, a large number of HA20 patients have been identified. HA20 patients present with variable clinical manifestations, even among family members carrying the same variant. These patients are initially diagnosed with diseases such as Behcet’s disease, juvenile idiopathic arthritis, rheumatoid arthritis, periodic fever with aphthous pharyngitis and adenitis, autoimmune thyroiditis, Crohn’s disease, SLE, or adult Still’s disease, along with cardiovascular symptoms, nephrotic syndrome, vasculitis, respiratory tract infections, and other diseases caused by autoantibodies (61).

Compared to other genes, CYLD variants have thus far only been linked to Brooke-Spiegler syndrome, familial cylindromatosis, and multiple familial piloepitheliomas (62). Patients with Brooke-Spiegler syndrome are predisposed to a range of skin adnexal tumors, such as cylindroma, trichoepithelioma, and spiradenoma. Familial cylindromatosis and multiple familial piloepitheliomas are also part of Brooke-Spiegler syndrome, with single lesions being the primary manifestation. However, due to the rarity of this disease, its mechanism has not been extensively studied.

OTULIN is a deubiquitinating enzyme that specifically hydrolyzes the M1 ubiquitin chain in RIPK1. Defects in the OTULIN gene have been shown to cause autoinflammation, panniculitis, and dermatological syndrome (AIPDS) (45). AIPDS is manifested as neutrophil infiltration and recurrent nodular panniculitis with recurrent fever, as well as increased immunoglobulin levels and autoantibody titers in the blood. OTULIN is the major deubiquitinating enzyme of the M1 ubiquitin chain and negatively regulates the NF-κB pathway (63). Stimulation of fibroblasts and PBMCs from OTULIN-deficient patients with TNF-a results in increased activation of NF-kB and MAPK and increased ubiquitination of M1 in RIPK1, suggesting that loss-of-function mutations in OTULIN lead to increased M1 ubiquitination, activation of TNF pathway and NF-κB-dependent inflammation (64).

## 5 Roles and mechanism of RIPK1 in the development of sepsis

**Figure 2:**
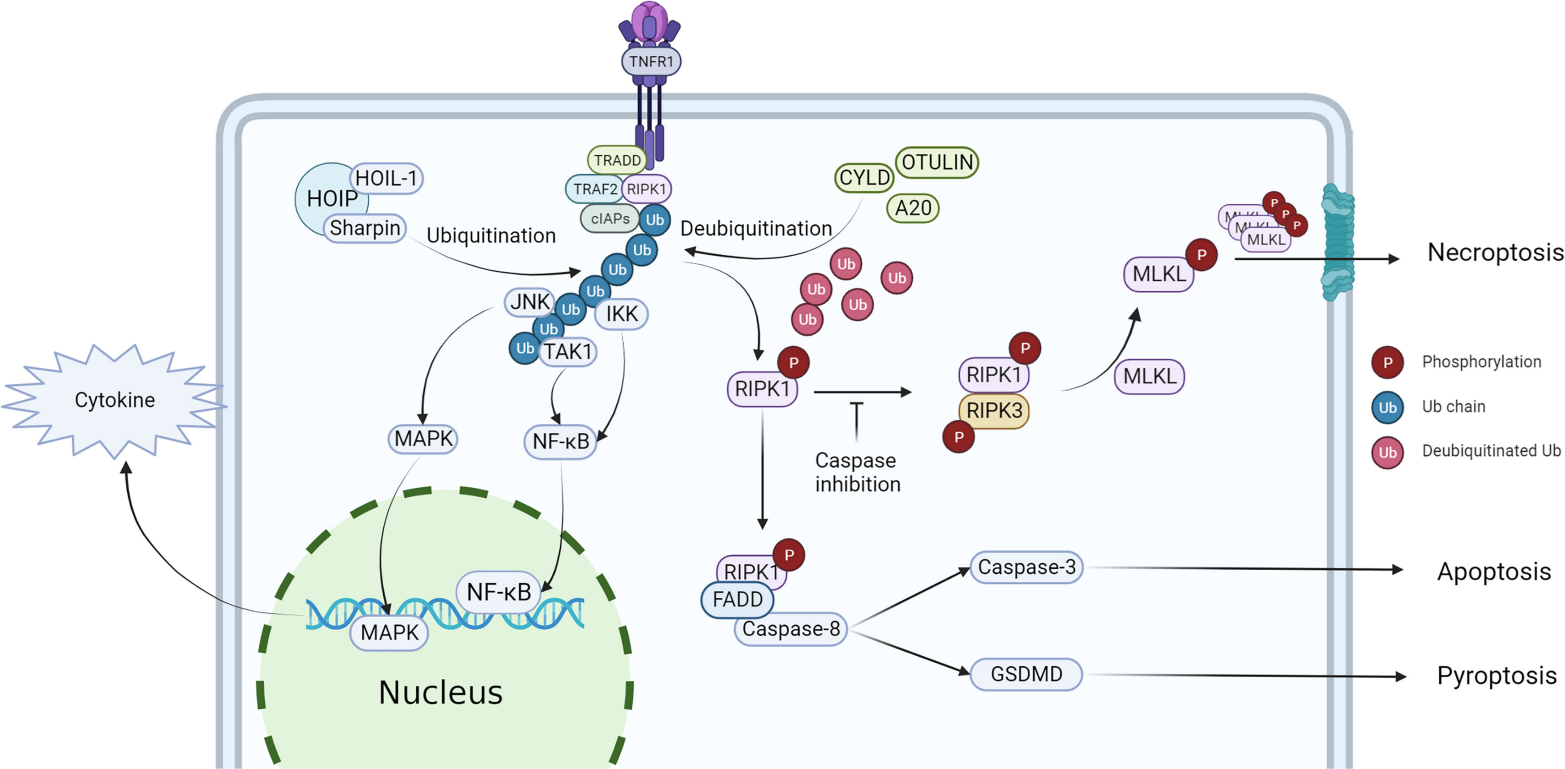
Regulation of RIPK1 and downstream effect. After stimulated by TNF-α, TRADD, TRAF2, RIPK1 and cIAPs would recruit to the intracellular terminus of TNFR1, then RIPK1 is ubiquitinated to form complex I, activating NF-κB and MAPK pathway. When RIPK1 has been deubiquitinated, the cell death pathway would be activated, triggering necroptosis, apoptosis and pyroptosis.

Studies have shown that both the kinase activity and scaffold structure of RIPK1 can induce inflammation, but while the scaffold structure only induces the transcription of cytokines, the kinase activity of RIPK1 induces cell death and a more intense inflammatory response (**Figure 2**). Consequently, the role of RIPK1 kinase activity in the systemic inflammatory response and sepsis has garnered increased attention. **Table 3** lists some animal experiments related to RIPK1 in sepsis. Caspase-8 inhibition is a crucial factor for RIPK1-induced necroptosis (69), and a variety of pathogens inhibit caspase-8 activity: Macrophages respond to infections with certain pathogens such as Serratia marcescens, Staphylococcus aureus, Streptococcus pneumoniae, Streptococcus monocytogenes, and uropathogenic E. coli by inhibiting caspase-8 activity, thereby inducing necrosome formation and necroptosis (70). Necroptosis, a complementary programmed death pathway that is activated when apoptosis and pyroptosis are inhibited. This process exposes immature intracellular pathogens to the tissue interstitial space and releases various chemokines that activate various immune cells for defense, which is beneficial for intracellular infections (71). However, severe infections can result in a large number of necroptosis-induced DAMPs and chemotaxis-induced neutrophil traps (NETs) that promote the release of cytokines, forming a positive feedback loop of the inflammatory response that ultimately leads to a systemic inflammatory response and sepsis. Recent studies have highlighted the role of RIPK1 kinase activity-induced necroptosis, which has been detected in vascular endothelial cells In recent years, the impact of RIPK1 kinase activity-induced necroptosis has gradually emerged in research. Studies have found that RIPK1-dependent necroptosis occurs in pulmonary vascular endothelial cells, and inhibition of necroptosis can alleviate the injury of ARDS induced by a large amount of LPS in a mouse model (72–74). Furthermore, RIPK3 knockdown can also reduce the severity of LPS-induced mouse ARDS (75).

**Table 3:** Study on RIPK1 and downstream sites in sepsis model.

Studies	Inducer	Species	Target Organs	Conclusion	Ref.
1	LPS	Mouse	Lung	Inhibition of necroptosis attenuates injury in subjects with ARDS	(74)
2	LPS	Mouse	Lung	RIPK3 knockdown attenuates ARDS-induced injury	(75)
3	LPS	Mouse	Lung	Necrotic apoptosis predominates upon stimulation with higher LPS doses rather than with lower doses	(75)
4	LPS	piglet	Intestine	The intestinal epithelial injury was attenuated by RIPK1 inhibition, and an increased level of MLKL phosphorylation was observed	(13)
5	LPS	piglet	Liver	Inhibition of RIPK1 with Nec-1 attenuates LPS-induced liver injury	(78)

Lipopolysaccharide (LPS) is a large molecule that is found in the outer membrane of gram-negative bacteria. The LPS-induced inflammatory response also contributes to the pathogenesis of sepsis. Toll-like receptor 4 (TLR4) recognizes LPS and activates downstream inflammatory response pathways (76), such as the MAPK and NF-κB pathways, through MyD88-dependent and MyD88-independent pathways. The MyD88-independent pathway requires the participation of RIPK1, which is recruited after the intracellular segment of TLR4 interacts with TRAF and immediately activates the inflammatory response pathway (77). The production of cytokines and DAMPs exacerbates the inflammatory response, ultimately inducing sepsis and organ dysfunction. Regulation of RIPK1 ubiquitination has potential value for the treatment of bacterial infections, but few studies have examined this mechanism (**Table 3**). In an LPS-induced septic piglet model, inhibition of RIPK1 attenuated intestinal epithelial injury, accompanied by an increase in the level of MLKL phosphorylation, suggesting that necroptosis affects intestinal epithelial cell death during sepsis (13). A similar result was observed in an LPS-induced liver injury model (78). In addition, necroptosis predominated in mice stimulated with high doses of LPS compared with low doses of LPS (75), and stimulation with various PAMPs enhanced necroptosis in macrophages expressing the RIPK1 K45A mutation (79). Wang et al. (80) found that upregulation of the deubiquitinating enzyme Cezanne in BV2 cells inhibited the NF-κB pathway, and inhibition of Cezanne was accompanied by increased levels of RIPK1 K63 ubiquitination. The importance of cell death in regulating systemic inflammation is highlighted by the RIPK1 knockout embryonic mice, which die during the embryonic stage due to systemic inflammation, and by blocking the necroptosis pathway and the MyD88 pathway reduce inflammation, but only mice with both RIPK3 and caspase-8 knockout survive after weaning (81).

In certain cases of pathogen infection, caspase8 activation can trigger pyroptosis by activating GSDMD (82). When caspase8 activity is inhibited, RIPK1 is phosphorylated and binds with RIPK3 to initiate necroptosis, providing an alternative route when pyroptosis is inhibited (69). For many years, caspase8 has been considered a key protein regulating cell death pathways, including apoptosis, pyroptosis, and necroptosis (69, 83). However, recent research has led to propose the concept of “inflammatory cell death” which integrates these pathways into a single entity known as “PANoptosis” (18, 84). At the core of PANoptosis is the PANoptosome, a complex of proteins comprising RIPK1, RIPK3, and caspase8 that form interactions through RHIM and DD domains with other related proteins to mediate pyroptosis and necroptosis (85). Prior research has shown that inhibition of RIPK1 can reduce inflammation, likely by disrupting PANoptosome formation. Therefore, the PANoptosome may serve as a critical complex in regulating inflammatory cell death (86, 87). However, the molecular mechanisms underlying its regulation of the inflammatory response are not yet fully understood, and further experimentation is needed to elucidate its therapeutic potential.

Despite the growing interest in sepsis models that focus on necroptosis or downstream regulators, the upstream pathways that regulate RIPK1 and its modifications have received comparatively less attention. Although several studies suggest that the deubiquitination of RIPK1 is a critical factor in the occurrence of necroptosis, direct evidence supporting this hypothesis remains limited. Recently, it has been proposed that cIAP2 downregulation and decreased ubiquitination of RIPK1 are evident in patients with H7N9-induced ARDS; however, changes in the ubiquitination and phosphorylation of RIPK1, or alterations in complex I, have yet to be fully elucidated (88).

## 6 Inhibitors of RIPK1 and its effects on sepsis

In clinical research, there has been relatively little investigation into the association between RIPK1 and sepsis (**Table 4**). Previous studies have suggested that serum RIPK1 does not differ significantly in relation to the severity of sepsis (95). Therefore, RIPK1 may not be suitable for determining the severity of sepsis. However, this research was based on the earlier sepsis 2.0 criteria, which differ from the currently widely used sepsis 3.0 criteria. Additionally, it did not evaluate other differences such as the degree of inflammatory response and organ function. Subsequent research has focused more on differences in indicators such as RIPK3 and MLKL (96, 97), but this does not necessarily indicate that inhibition of RIPK1 is ineffective in treating sepsis. The differences observed in RIPK3 and MLKL may be due to their location downstream in the pathway, which amplifies signals and makes them more likely to show differences. In recent years, the COVID-19 pandemic, with its prominent inflammatory response, has led some researchers to focus on RIPK1. In one study, respiratory epithelial cells collected from throat smears of symptomatic patients who had tested positive for SARS-CoV-2 by PCR showed positive results for phosphorylated RIPK1, while no phosphor-RIPK1-positive cells were detected in control samples from healthy individuals (91). Another study of critically ill COVID-19 patients found significantly higher levels of serum RIPK1 than in healthy controls, with differences observed in relation to disease severity (98).

**Table 4:** clinical trial of RIPK1.

Studies	Disease	Conclusion	Ref.
1	Sepsis	No difference in RIPK1 in sepsis2.0 patients	(95)
2	COVID-19 infection	Compares to healthy control immunohistochemical analyses of all epithelial cell samples from the COVID-19 patients were positive for active phosphorylated RIPK1	(91)
3	Critical COVID-19 patients	RIPK3, MLKL, HMGB1, and RIPK1 plasma concentrations of patients with severe COVID-19 were higher than those of moderate patients	(98)
4	ventilator-induced lung injury	Elevation of plasma RIPK3, but not RIPK1 or MLKL, in mechanically ventilated patients	(99)

Given the exploration of RIPK1 in rheumatoid immune diseases and inflammatory reactions, there have been studies using RIPK1 inhibitors to regulate the inflammatory response in sepsis (**Table 5**). The traditional RIPK1 inhibitors, necrostatin-1 (NEC-1) and RIPA56 have been widely used in various animal inflammation models, both of which can reduce the inflammatory response and decrease necrotic apoptosis (23, 89, 90): in the TNF-α-induced SIRS model, the use of NEC-1 was found to alleviate the inflammatory response and reduce the mortality rate of mice (23).

**Table 5:** RIPK1 inhibitors and their effect.

Studies	Drug	Species	Model	Conclusion	Ref.
1	Nec-1	Mice	TNF induced SIRS	Nec-1 Administration Prevents Mortality, Cell Death, and Sustained Inflammation Associated with TNF-Induced SIRS	(23)
2	Nec-1	Rat	oleic acid-induced acute respiratory distress syndrome	Nec-1 pretreatment reduced necroptosis by inhibiting RIPK1- RIPK3-MLKL pathway in OA-induced ARDS and decreased aggregation of inflammatory cells and TNF-a level in BALF	(89)
3	Nec-1; RIPA56	Rat	LPS/GalN induced acute on chronic liver failure	NEC-1 treatment prevents the occurrence of ACLF, and RIPA56 prevents RIPK1/RIPK3 mediated necroptosis	(90)
4	primidone	C57BL/6JRj; MEF; Cell lines	TNF induced SIRS	Primidone can block the activity of RIPK1 in various cell types, preventing cell death and reducing the release of pro-inflammatory cytokines. Improves TNFα-treated mice hypothermia, lung injury and mortality	(91)
5	ZB-R-55	Mouse	LPS/TNF-induced sepsis	ZB-R-55, administered orally at 10 mg/kg, showed protection from hypothermia, cytokine storm and survival rate.	(92)
6	SAR443122	Homo Spain	COVID-19 infection	No result publication	(93)
7	DNL104	Homo Spain	TNF -treated PBMC from healthy donors	DNL104 can reduce phosphorylation of RIPK1 in TNF+zVAD treated PBMC.	(94)

Subsequently, in the oleic acid-induced ARDS model, NEC-1 was also found to improve the oxygenation index of ARDS mice (89). In the mouse model of acute on chronic liver failure, using LPS to stimulate chronic liver failure mice, it was found that RIPK1-mediated cell death in liver cells could be induced, and the use of NEC-1 could alleviate the degree of acute liver failure. Furthermore, the use of the RIPK1 inhibitor, RIIPA56, which is more selective for RIPK1, led to a decrease in the expression of RIPK3 and the phosphorylation of MLKL, significantly reducing necrotic apoptosis in liver cells (90). Moreover, primidone, a drug previously used to treat epilepsy, has been found to have an inhibitory effect on RIPK1. By inhibiting the formation of complex II, primidone can reduce the occurrence of necrotic apoptosis in cells under TNF stimulation. Further animal experiments have found that primidone can alleviate renal ischemia-reperfusion injury, reduce necrotic apoptosis in the renal outer medulla, protect renal function, and also reduce the mortality rate of mice mediated by TNF-α (91). In terms of new drug development, ZB-R-55 has improved its molecular structure, pharmacokinetics, and other aspects, achieving a more efficient RIPK1 inhibitory effect. In inhibiting the cytokine storm, it has shown a similar effect to dexamethasone (92). In addition, there is a clinical trial using RIPK1 inhibitors for COVID-19 infected patients. The trial has collected data, but the results have not yet been published (93).

## 7 Discussion and perspectives

Sepsis is one of the most difficult clinical challenges. The mechanism by which the initial infection leads to excessive inflammatory responses remains unclear, but interventions targeting cytokine storms may be used as one of the treatments to reduce organ damage in individuals with sepsis, such as the use of glucocorticoids to reduce inflammatory responses, but the benefits are limited (100). Hemoperfusion and other approaches do not produce the ideal therapeutic effect, suggesting that perhaps interventions targeting cytokines at more upstream sites in the pathway will achieve better results. The initial immune response after pathogen invasion that causes the subsequent exacerbation of the inflammatory response is one of the theoretical bases of the cytokine storm, and the blockade of waterfall activation of the cytokine storm may be an ideal target, while the regulation of RIPK1 controls the direction of cellular outcomes after TNF-a stimulation.

Previous clinical studies have primarily focused on measuring the concentration of serum RIPK1, providing a reference for predicting the occurrence and development of sepsis. However, the advantages of RIPK1 in this direction seem to be less significant compared to downstream proteins such as RIPK3 and MLKL. From another perspective, it may be more suitable as an intervention target. As a critical node in necroptosis, apoptosis, and necroptosis, RIPK1 regulates the process of inflammatory cell death and thereby affects the course of the inflammatory response. One clear thing is that inhibiting RIPK1 can reduce RIPK3 and MLKL phosphorylation, as well as decrease the release of inflammatory factors such as HMGB-1, thus reducing the degree of the inflammatory response. In fact, it even has an effect comparable to that of dexamethasone (92). Therefore, RIPK1 inhibition may be another means of alleviating the cytokine storm associated with sepsis.

However, studies of RIPK1 in sepsis are still lacking. The effect of treatments targeting RIPK1 on the development of sepsis has not been clarified. Fortunately, there has been a growing body of research aimed at exploring the role of RIPK1 in regulating the inflammatory response in sepsis. This shift towards targeting RIPK1 function has spurred the development of numerous drugs, which are currently being evaluated in clinical trials. Thus, studies examining the regulation of RIPK1 may hopefully provide a better understanding of the mechanism of sepsis and directions for the treatment of sepsis in the future.

## Author contributions

XL: Investigation, writing - original drift. A-LT: Writing – review. JC: Resources, visualization. NG: Acquisition of data. GZ: Conceptualization. CX: Supervision. All authors contributed to the article and approved the submitted version. 

## References

[1] Degterev A Huang Z Boyce M Li Y Jagtap P Mizushima N. Chemical inhibitor of nonapoptotic cell death with therapeutic potential for ischemic brain injury. Nat Chem Biol (2005) 1:112–9. doi: 10.1038/nchembio711 16408008

[2] Holler N Zaru R Micheau O Thome M Attinger A Valitutti S. Fas triggers an alternative, caspase-8–independent cell death pathway using the kinase RIP as effector molecule. Nat Immunol (2000) 1:489–95. doi: 10.1038/82732 11101870

[3] He S Wang X. RIP kinases as modulators of inflammation and immunity. Nat Immunol (2018) 19:912–22. doi: 10.1038/s41590-018-0188-x 30131615

[4] Ofengeim D Yuan J. Regulation of RIP1 kinase signalling at the crossroads of inflammation and cell death. Nat Rev Mol Cell Biol (2013) 14:727–36. doi: 10.1038/nrm3683 24129419

[5] Sun X Yin J Starovasnik MA Fairbrother WJ Dixit VM. Identification of a novel homotypic interaction motif required for the phosphorylation of receptor-interacting protein (RIP) by RIP3 *. J Biol Chem (2002) 277:9505–11. doi: 10.1074/jbc.M109488200 11734559

[6] Hsu H Huang J Shu H-B Baichwal V Goeddel DV. TNF-dependent recruitment of the protein kinase RIP to the TNF receptor-1 signaling complex. Immunity (1996) 4:387–96. doi: 10.1016/S1074-7613(00)80252-6 8612133

[7] Berghe TV Linkermann A Jouan-Lanhouet S Walczak H Vandenabeele P. Regulated necrosis: the expanding network of non-apoptotic cell death pathways. Nat Rev Mol Cell Biol (2014) 15:135–47. doi: 10.1038/nrm3737 24452471

[8] Micheau O Tschopp J. Induction of TNF receptor I-mediated apoptosis *via* two sequential signaling complexes. Cell (2003) 114:181–90. doi: 10.1016/S0092-8674(03)00521-X 12887920

[9] Jang K-H Jang T Son E Choi S Kim E. Kinase-independent role of nuclear RIPK1 in regulating parthanatos through physical interaction with PARP1 upon oxidative stress. Biochim Biophys Acta BBA - Mol Cell Res (2018) 1865:132–41. doi: 10.1016/j.bbamcr.2017.10.004 28993228

[10] Ramnarain DB Paulmurugan R Park S Mickey BE Asaithamby A Saha D. RIP1 links inflammatory and growth factor signaling pathways by regulating expression of the EGFR. Cell Death Differ (2008) 15:344–53. doi: 10.1038/sj.cdd.4402268 18007664

[11] Weber K Roelandt R Bruggeman I Estornes Y Vandenabeele P. Nuclear RIPK3 and MLKL contribute to cytosolic necrosome formation and necroptosis. Commun Biol (2018) 1:6. doi: 10.1038/s42003-017-0007-1 30271893 PMC6123744

[12] Yoon S Bogdanov K Kovalenko A Wallach D. Necroptosis is preceded by nuclear translocation of the signaling proteins that induce it. Cell Death Differ (2016) 23:253–60. doi: 10.1038/cdd.2015.92 PMC4716306 26184911

[13] Liu Y Xu Q Wang Y Liang T Li X Wang D. Necroptosis is active and contributes to intestinal injury in a piglet model with lipopolysaccharide challenge. Cell Death Dis (2021) 12:62. doi: 10.1038/s41419-020-03365-1 33431831 PMC7801412

[14] Feoktistova M Makarov R Brenji S Schneider AT Hooiveld GJ Luedde T. A20 promotes ripoptosome formation and TNF-induced apoptosis *via* cIAPs regulation and NIK stabilization in keratinocytes. Cells (2020) 9:E351. doi: 10.3390/cells9020351 PMC7072579 32028675

[15] Yuan J Amin P Ofengeim D. Necroptosis and RIPK1-mediated neuroinflammation in CNS diseases. Nat Rev Neurosci (2019) 20:19–33. doi: 10.1038/s41583-018-0093-1 30467385 PMC6342007

[16] DeRoo E Zhou T Liu B. The role of RIPK1 and RIPK3 in cardiovascular disease. Int J Mol Sci (2020) 21:E8174. doi: 10.3390/ijms21218174 PMC7663726 33142926

[17] Ofengeim D Ito Y Najafov A Zhang Y Shan B DeWitt JP. Activation of necroptosis in multiple sclerosis. Cell Rep (2015) 10:1836–49. doi: 10.1016/j.celrep.2015.02.051 PMC4494996 25801023

[18] Malireddi RKS Gurung P Kesavardhana S Samir P Burton A Mummareddy H. Innate immune priming in the absence of TAK1 drives RIPK1 kinase activity-independent pyroptosis, apoptosis, necroptosis, and inflammatory disease. J Exp Med (2020) 217:e20191644. doi: 10.1084/jem.20191644 PMC7062518 31869420

[19] Slebos D-J Ryter SW van der Toorn M Liu F Guo F Baty CJ. Mitochondrial localization and function of heme oxygenase-1 in cigarette smoke-induced cell death. Am J Respir Cell Mol Biol (2007) 36:409–17. doi: 10.1165/rcmb.2006-0214OC PMC1899328 17079780

[20] Mizumura K Cloonan SM Nakahira K Bhashyam AR Cervo M Kitada T. Mitophagy-dependent necroptosis contributes to the pathogenesis of COPD. J Clin Invest (2014) 124:3987–4003. doi: 10.1172/JCI74985 25083992 PMC4151233

[21] Li Y Führer M Bahrami E Socha P Klaudel-Dreszler M Bouzidi A. Human RIPK1 deficiency causes combined immunodeficiency and inflammatory bowel diseases. Proc Natl Acad Sci USA. (2019) 116:970–5. doi: 10.1073/pnas.1813582116 PMC6338855 30591564

[22] Cuchet-Lourenço D Eletto D Wu C Plagnol V Papapietro O Curtis J. Biallelic RIPK1 mutations in humans cause severe immunodeficiency, arthritis, and intestinal inflammation. Science (2018) 361:810–3. doi: 10.1126/science.aar2641 PMC6529353 30026316

[23] Duprez L Takahashi N Van Hauwermeiren F Vandendriessche B Goossens V Vanden Berghe T. RIP kinase-dependent necrosis drives lethal systemic inflammatory response syndrome. Immunity (2011) 35:908–18. doi: 10.1016/j.immuni.2011.09.020 22195746

[24] Dillon CP Weinlich R Rodriguez DA Cripps JG Quarato G Gurung P. RIPK1 blocks early postnatal lethality mediated by caspase-8 and RIPK3. Cell (2014) 157:1189–202. doi: 10.1016/j.cell.2014.04.018 PMC4068710 24813850

[25] Wang Q Fan D Xia Y Ye Q Xi X Zhang G. The latest information on the RIPK1 post-translational modifications and functions. BioMed Pharmacother (2021) 142:112082. doi: 10.1016/j.biopha.2021.112082 34449307

[26] Kaiser WJ Daley-Bauer LP Thapa RJ Mandal P Berger SB Huang C. RIP1 suppresses innate immune necrotic as well as apoptotic cell death during mammalian parturition. Proc Natl Acad Sci U.S.A. (2014) 111:7753–8. doi: 10.1073/pnas.1401857111 PMC4040608 24821786

[27] Newton K Dugger DL Wickliffe KE Kapoor N de Almagro MC Vucic D. Activity of protein kinase RIPK3 determines whether cells die by necroptosis or apoptosis. Science (2014) 343:1357–60. doi: 10.1126/science.1249361 24557836

[28] Stark K Goncharov T Varfolomeev E Xie L Ngu H Peng I. Genetic inactivation of RIP1 kinase activity in rats protects against ischemic brain injury. Cell Death Dis (2021) 12:1–15. doi: 10.1038/s41419-021-03651-6 33828080 PMC8026634

[29] Newton K Dugger DL Maltzman A Greve JM Hedehus M Martin-McNulty B. RIPK3 deficiency or catalytically inactive RIPK1 provides greater benefit than MLKL deficiency in mouse models of inflammation and tissue injury. Cell Death Differ (2016) 23:1565–76. doi: 10.1038/cdd.2016.46 PMC5072432 27177019

[30] Zhang Y Su SS Zhao S Yang Z Zhong C-Q Chen X. RIP1 autophosphorylation is promoted by mitochondrial ROS and is essential for RIP3 recruitment into necrosome. Nat Commun (2017) 8:14329. doi: 10.1038/ncomms14329 28176780 PMC5309790

[31] Laurien L Nagata M Schünke H Delanghe T Wiederstein JL Kumari S. Autophosphorylation at serine 166 regulates RIP kinase 1-mediated cell death and inflammation. Nat Commun (2020) 11:1747. doi: 10.1038/s41467-020-15466-8 32269263 PMC7142081

[32] Dondelinger Y Delanghe T Priem D Wynosky-Dolfi MA Sorobetea D Rojas-Rivera D. Serine 25 phosphorylation inhibits RIPK1 kinase-dependent cell death in models of infection and inflammation. Nat Commun (2019) 10:1729. doi: 10.1038/s41467-019-09690-0 30988283 PMC6465317

[33] Du J Xiang Y Liu H Liu S Kumar A Xing C. RIPK1 dephosphorylation and kinase activation by PPP1R3G/PP1γ promote apoptosis and necroptosis. Nat Commun (2021) 12:7067. doi: 10.1038/s41467-021-27367-5 34862394 PMC8642546

[34] Lafont E Draber P Rieser E Reichert M Kupka S de Miguel D. TBK1 and IKKϵ prevent TNF-induced cell death by RIPK1 phosphorylation. Nat Cell Biol (2018) 20:1389–99. doi: 10.1038/s41556-018-0229-6 PMC6268100 30420664

[35] Jaco I Annibaldi A Lalaoui N Wilson R Tenev T Laurien L. MK2 phosphorylates RIPK1 to prevent TNF-induced cell death. Mol Cell (2017) 66:698–710.e5. doi: 10.1016/j.molcel.2017.05.003 28506461 PMC5459754

[36] Menon MB Gropengießer J Fischer J Novikova L Deuretzbacher A Lafera J. p38MAPK/MK2-dependent phosphorylation controls cytotoxic RIPK1 signalling in inflammation and infection. Nat Cell Biol (2017) 19:1248–59. doi: 10.1038/ncb3614 28920954

[37] Geng J Ito Y Shi L Amin P Chu J Ouchida AT. Regulation of RIPK1 activation by TAK1-mediated phosphorylation dictates apoptosis and necroptosis. Nat Commun (2017) 8:359. doi: 10.1038/s41467-017-00406-w 28842570 PMC5572456

[38] Kist M Kőműves LG Goncharov T Dugger DL Yu C Roose-Girma M. Impaired RIPK1 ubiquitination sensitizes mice to TNF toxicity and inflammatory cell death. Cell Death Differ (2021) 28:985–1000. doi: 10.1038/s41418-020-00629-3 32999468 PMC7937686

[39] de Almagro MC Goncharov T Izrael-Tomasevic A Duttler S Kist M Varfolomeev E. Coordinated ubiquitination and phosphorylation of RIP1 regulates necroptotic cell death. Cell Death Differ (2017) 24:26–37. doi: 10.1038/cdd.2016.78 27518435 PMC5260504

[40] He M Wang M Huang Y Peng W Zheng Z Xia N. The ORF3 protein of genotype 1 hepatitis e virus suppresses TLR3-induced NF-κB signaling *via* TRADD and RIP1. Sci Rep (2016) 6:27597. doi: 10.1038/srep27597 27270888 PMC4897786

[41] Meng H Liu Z Li X Wang H Jin T Wu G. Death-domain dimerization-mediated activation of RIPK1 controls necroptosis and RIPK1-dependent apoptosis. Proc Natl Acad Sci (2018) 115:E2001–9. doi: 10.1073/pnas.1722013115 PMC5834731 29440439

[42] Li X Zhang M Huang X Liang W Li G Lu X. Ubiquitination of RIPK1 regulates its activation mediated by TNFR1 and TLRs signaling in distinct manners. Nat Commun (2020) 11:6364. doi: 10.1038/s41467-020-19935-y 33311474 PMC7733462

[43] Tu H Tang Y Zhang J Cheng L Joo D Zhao X. Linear ubiquitination of RIPK1 on Lys612 regulates systemic inflammation *via* preventing cell death. J Immunol (2021) 207:602–12. doi: 10.4049/jimmunol.2100299 34162724

[44] Keusekotten K Elliott PR Glockner L Fiil BK Damgaard RB Kulathu Y. OTULIN antagonizes LUBAC signaling by specifically hydrolyzing Met1-linked polyubiquitin. Cell (2013) 153:1312–26. doi: 10.1016/j.cell.2013.05.014 PMC3690481 23746843

[45] Damgaard RB Walker JA Marco-Casanova P Morgan NV Titheradge HL Elliott PR. The deubiquitinase OTULIN is an essential negative regulator of inflammation and autoimmunity. Cell (2016) 166:1215–1230.e20. doi: 10.1016/j.cell.2016.07.019 27523608 PMC5002269

[46] Mahoney DJ Cheung HH Mrad RL Plenchette S Simard C Enwere E. Both cIAP1 and cIAP2 regulate TNFalpha-mediated NF-kappaB activation. Proc Natl Acad Sci U.S.A. (2008) 105:11778–83. doi: 10.1073/pnas.0711122105 PMC2575330 18697935

[47] Zhang J Webster JD Dugger DL Goncharov T Roose-Girma M Hung J. Ubiquitin ligases cIAP1 and cIAP2 limit cell death to prevent inflammation. Cell Rep (2019) 27:2679–2689.e3. doi: 10.1016/j.celrep.2019.04.111 31141691

[48] Wong WW-L Vince JE Lalaoui N Lawlor KE Chau D Bankovacki A. cIAPs and XIAP regulate myelopoiesis through cytokine production in an RIPK1- and RIPK3-dependent manner. Blood (2014) 123:2562–72. doi: 10.1182/blood-2013-06-510743 24497535

[49] Liu F-Q. Analysis of differentially expressed genes in rheumatoid arthritis and osteoarthritis by integrated microarray analysis. J Cell Biochem (2019) 120:12653–64. doi: 10.1002/jcb.28533 30834598

[50] Nguyen VQ Eden K Morrison HA Sammons MB Knight KK Sorrentino S. Noncanonical NF-κB signaling upregulation in inflammatory bowel disease patients is associated with loss of response to anti-TNF agents. Front Pharmacol (2021) 12:655887. doi: 10.3389/fphar.2021.655887 34177575 PMC8223059

[51] Ekedahl J Bertrand J Grigoriev MY Muller M Magnusson C Lewensohn R. Expression of inhibitor of apoptosis proteins in small- and non-small-cell lung carcinoma cells. Exp Cell Res (2002) 279:277–90. doi: 10.1006/excr.2002.5608 12243753

[52] Oda H Beck DB Kuehn HS Sampaio Moura N Hoffmann P Ibarra M. Second case of HOIP deficiency expands clinical features and defines inflammatory transcriptome regulated by LUBAC. Front Immunol (2019) 10:479. doi: 10.3389/fimmu.2019.00479 30936877 PMC6431612

[53] Rickard JA Anderton H Etemadi N Nachbur U Darding M Peltzer N. TNFR1-dependent cell death drives inflammation in sharpin-deficient mice. eLife (2014) 3:e03464. doi: 10.7554/eLife.03464 25443632 PMC4270099

[54] Peltzer N Darding M Montinaro A Draber P Draberova H Kupka S. LUBAC is essential for embryogenesis by preventing cell death and enabling haematopoiesis. Nature (2018) 557:112–7. doi: 10.1038/s41586-018-0064-8 PMC5947819 29695863

[55] Kumari S Redouane Y Lopez-Mosqueda J Shiraishi R Romanowska M Lutzmayer S. Sharpin prevents skin inflammation by inhibiting TNFR1-induced keratinocyte apoptosis. eLife (2014) 3:e03422. doi: 10.7554/eLife.03422 25443631 PMC4225491

[56] Peltzer N Rieser E Taraborrelli L Draber P Darding M Pernaute B. HOIP deficiency causes embryonic lethality by aberrant TNFR1-mediated endothelial cell death. Cell Rep (2014) 9:153–65. doi: 10.1016/j.celrep.2014.08.066 25284787

[57] Adrianto I Wen F Templeton A Wiley G King JB Lessard CJ. Association of a functional variant downstream of TNFAIP3 with systemic lupus erythematosus. Nat Genet (2011) 43:253–8. doi: 10.1038/ng.766 PMC3103780 21336280

[58] Wang S Wen F Tessneer KL Gaffney PM. TALEN-mediated enhancer knockout influences TNFAIP3 gene expression and mimics a molecular phenotype associated with systemic lupus erythematosus. Genes Immun (2016) 17:165–70. doi: 10.1038/gene.2016.4 PMC4840072 26821284

[59] Wang S Wen F Wiley GB Kinter MT Gaffney PM. An enhancer element harboring variants associated with systemic lupus erythematosus engages the TNFAIP3 promoter to influence A20 expression. PloS Genet (2013) 9:e1003750. doi: 10.1371/journal.pgen.1003750 24039598 PMC3764111

[60] Zhou Q Wang H Schwartz DM Stoffels M Park YH Zhang Y. Loss-of-function mutations in TNFAIP3 leading to A20 haploinsufficiency cause an early-onset autoinflammatory disease. Nat Genet (2016) 48:67–73. doi: 10.1038/ng.3459 26642243 PMC4777523

[61] Yu M-P Xu X-S Zhou Q Deuitch N Lu M-P. Haploinsufficiency of A20 (HA20): updates on the genetics, phenotype, pathogenesis and treatment. World J Pediatr WJP (2020) 16:575–84. doi: 10.1007/s12519-019-00288-6 31587140

[62] Young AL Kellermayer R Szigeti R Tészás A Azmi S Celebi JT. CYLD mutations underlie brooke-spiegler, familial cylindromatosis, and multiple familial trichoepithelioma syndromes. Clin Genet (2006) 70:246–9. doi: 10.1111/j.1399-0004.2006.00667.x 16922728

[63] Zhao M Song K Hao W Wang L Patil G Li Q. Non-proteolytic ubiquitination of OTULIN regulates NF-κB signaling pathway. J Mol Cell Biol (2020) 12:163–75. doi: 10.1093/jmcb/mjz081 PMC7181720 31504727

[64] Zhou Q Yu X Demirkaya E Deuitch N Stone D Tsai WL. Biallelic hypomorphic mutations in a linear deubiquitinase define otulipenia, an early-onset autoinflammatory disease. Proc Natl Acad Sci U.S.A. (2016) 113:10127–32. doi: 10.1073/pnas.1612594113 PMC5018768 27559085

[65] Polykratis A Hermance N Zelic M Roderick J Kim C Van T-M. RIPK1 kinase inactive mice are viable and protected from TNF-induced necroptosis *in vivo* . J Immunol Baltim Md 1950 (2014) 193:1539–43. doi: 10.4049/jimmunol.1400590 PMC4119562 25015821

[66] Tang Y Tu H Zhang J Zhao X Wang Y Qin J. K63-linked ubiquitination regulates RIPK1 kinase activity to prevent cell death during embryogenesis and inflammation. Nat Commun (2019) 10:4157. doi: 10.1038/s41467-019-12033-8 31519887 PMC6744441

[67] Boisson B Laplantine E Dobbs K Cobat A Tarantino N Hazen M. Human HOIP and LUBAC deficiency underlies autoinflammation, immunodeficiency, amylopectinosis, and lymphangiectasia. J Exp Med (2015) 212:939–51. doi: 10.1084/jem.20141130 PMC4451137 26008899

[68] Damgaard RB Elliott PR Swatek KN Maher ER Stepensky P Elpeleg O. OTULIN deficiency in ORAS causes cell type-specific LUBAC degradation, dysregulated TNF signalling and cell death. EMBO Mol Med (2019) 11:e9324. doi: 10.15252/emmm.201809324 30804083 PMC6404114

[69] Fritsch M Günther SD Schwarzer R Albert M-C Schorn F Werthenbach JP. Caspase-8 is the molecular switch for apoptosis, necroptosis and pyroptosis. Nature (2019) 575:683–7. doi: 10.1038/s41586-019-1770-6 31748744

[70] González-Juarbe N Gilley RP Hinojosa CA Bradley KM Kamei A Gao G. Pore-forming toxins induce macrophage necroptosis during acute bacterial pneumonia. PloS Pathog (2015) 11:e1005337. doi: 10.1371/journal.ppat.1005337 26659062 PMC4676650

[71] Weng D Marty-Roix R Ganesan S Proulx MK Vladimer GI Kaiser WJ. Caspase-8 and RIP kinases regulate bacteria-induced innate immune responses and cell death. Proc Natl Acad Sci U.S.A. (2014) 111:7391–6. doi: 10.1073/pnas.1403477111 PMC4034196 24799678

[72] Zelic M Roderick JE O’Donnell JA Lehman J Lim SE Janardhan HP. RIP kinase 1-dependent endothelial necroptosis underlies systemic inflammatory response syndrome. J Clin Invest (2018) 128:2064–75. doi: 10.1172/JCI96147 PMC5919800 29664014

[73] Wang L Zhou L Zhou Y Liu L Jiang W Zhang H. Necroptosis in pulmonary diseases: A new therapeutic target. Front Pharmacol (2021) 12:737129. doi: 10.3389/fphar.2021.737129 34594225 PMC8476758

[74] Yu X Mao M Liu X Shen T Li T Yu H. A cytosolic heat shock protein 90 and co-chaperone p23 complex activates RIPK3/MLKL during necroptosis of endothelial cells in acute respiratory distress syndrome. J Mol Med Berl Ger (2020) 98:569–83. doi: 10.1007/s00109-020-01886-y 32072232

[75] Wang L Wang T Li H Liu Q Zhang Z Xie W. Receptor interacting protein 3-mediated necroptosis promotes lipopolysaccharide-induced inflammation and acute respiratory distress syndrome in mice. PloS One (2016) 11:e0155723. doi: 10.1371/journal.pone.0155723 27195494 PMC4873150

[76] Murad S. Toll-like receptor 4 in inflammation and angiogenesis: A double-edged sword. Front Immunol (2014) 5:313. doi: 10.3389/fimmu.2014.00313 25071774 PMC4083339

[77] Bohannon JK Hernandez A Enkhbaatar P Adams WL Sherwood ER. The immunobiology of toll-like receptor 4 agonists: From endotoxin tolerance to immunoadjuvants. Shock (2013) 40:451–62. doi: 10.1097/SHK.0000000000000042 PMC3919163 23989337

[78] Xu Q Guo J Li X Wang Y Wang D Xiao K. Necroptosis underlies hepatic damage in a piglet model of lipopolysaccharide-induced sepsis. Front Immunol (2021) 12:633830. doi: 10.3389/fimmu.2021.633830 33777021 PMC7994362

[79] Shutinoski B Alturki NA Rijal D Bertin J Gough PJ Schlossmacher MG. K45A mutation of RIPK1 results in poor necroptosis and cytokine signaling in macrophages, which impacts inflammatory responses. vivo. Cell Death Differ (2016) 23:1628–37. doi: 10.1038/cdd.2016.51 PMC5041191 27258786

[80] Wang Z-C Chen Q Wang J Yu L-S Chen L-W. Sulforaphane mitigates LPS-induced neuroinflammation through modulation of Cezanne/NF-κB signalling. Life Sci (2020) 262:118519. doi: 10.1016/j.lfs.2020.118519 33010279

[81] Rickard JA O’Donnell JA Evans JM Lalaoui N Poh AR Rogers T. RIPK1 regulates RIPK3-MLKL-driven systemic inflammation and emergency hematopoiesis. Cell (2014) 157:1175–88. doi: 10.1016/j.cell.2014.04.019 24813849

[82] Zheng Z Deng W Bai Y Miao R Mei S Zhang Z. The lysosomal rag-ragulator complex licenses RIPK1 and caspase-8-mediated pyroptosis by yersinia. Science (2021) 372:eabg0269. doi: 10.1126/science.abg0269 35058659 PMC8769499

[83] Bertheloot D Latz E Franklin BS. Necroptosis, pyroptosis and apoptosis: an intricate game of cell death. Cell Mol Immunol (2021) 18:1106–21. doi: 10.1038/s41423-020-00630-3 PMC8008022 33785842

[84] Malireddi RKS Kesavardhana S Kanneganti T-D. ZBP1 and TAK1: Master regulators of NLRP3 Inflammasome/Pyroptosis, apoptosis, and necroptosis (PAN-optosis). Front Cell Infect Microbiol (2019) 9:406. doi: 10.3389/fcimb.2019.00406 31850239 PMC6902032

[85] Malireddi RKS Kesavardhana S Karki R Kancharana B Burton AR Kanneganti T-D. RIPK1 distinctly regulates yersinia-induced inflammatory cell death, PANoptosis. ImmunoHorizons (2020) 4:789–96. doi: 10.4049/immunohorizons.2000097 PMC7906112 33310881

[86] Samir P Malireddi RKS Kanneganti T-D. The PANoptosome: A deadly protein complex driving pyroptosis, apoptosis, and necroptosis (PANoptosis). Front Cell Infect Microbiol (2020) 10:238. doi: 10.3389/fcimb.2020.00238 32582562 PMC7283380

[87] Wang Y Kanneganti T-D. From pyroptosis, apoptosis and necroptosis to PANoptosis: A mechanistic compendium of programmed cell death pathways. Comput Struct Biotechnol J (2021) 19:4641–57. doi: 10.1016/j.csbj.2021.07.038 PMC8405902 34504660

[88] Qin C Sai X-Y Qian X-F Wu Y Zou L-F Wang H-M. Close relationship between cIAP2 and human ARDS induced by severe H7N9 infection. BioMed Res Int (2019) 2019:2121357. doi: 10.1155/2019/2121357 31080811 PMC6475567

[89] Pan L Yao D-C Yu Y-Z Li S-J Chen B-J Hu G-H. Necrostatin-1 protects against oleic acid-induced acute respiratory distress syndrome in rats. Biochem Biophys Res Commun (2016) 478:1602–8. doi: 10.1016/j.bbrc.2016.08.163 27586277

[90] Kondo T Macdonald S Engelmann C Habtesion A Macnaughtan J Mehta G. The role of RIPK1 mediated cell death in acute on chronic liver failure. Cell Death Dis (2021) 13:1–12. doi: 10.1038/s41419-021-04442-9 34921136 PMC8683430

[91] Riebeling T Jamal K Wilson R Kolbrink B von Samson-Himmelstjerna FA Moerke C. Primidone blocks RIPK1-driven cell death and inflammation. Cell Death Differ (2021) 28:1610–26. doi: 10.1038/s41418-020-00690-y PMC7712602 33273695

[92] Yang X Lu H Xie H Zhang B Nie T Fan C. Potent and selective RIPK1 inhibitors targeting dual-pockets for the treatment of systemic inflammatory response syndrome and sepsis. Angew Chem Int Ed Engl (2022) 61:e202114922. doi: 10.1002/anie.202114922 34851543

[93] Clot P-F Farenc C Suratt B Krahnke T Tardat A Florian P. Late breaking abstract - immunomodulatory and clinical effects of RIPK1 inhibitor SAR443122 in patients with severe COVID-19. Eur Respir J (2021) 58:PA2363. doi: 10.1183/13993003.congress-2021.PA2363 PMC10903152 38419035

[94] Grievink HW Heuberger JAAC Huang F Chaudhary R Birkhoff WAJ Tonn GR. DNL104, a centrally penetrant RIPK1 inhibitor, inhibits RIP1 kinase phosphorylation in a randomized phase I ascending dose study in healthy volunteers. Clin Pharmacol Ther (2020) 107:406–14. doi: 10.1002/cpt.1615 31437302

[95] Wang B Li J Gao H-M Xing Y-H Lin Z Li H-J. Necroptosis regulated proteins expression is an early prognostic biomarker in patient with sepsis: a prospective observational study. Oncotarget (2017) 8:84066–73. doi: 10.18632/oncotarget.21099 PMC5663577 29137405

[96] Mallarpu CS Ponnana M Prasad S Singarapu M Kim J Haririparsa N. Distinct cell death markers identified in critical care patient survivors diagnosed with sepsis. Immunol Lett (2021) 231:1–10. doi: 10.1016/j.imlet.2020.12.009 33406390

[97] Yoo H Im Y Ko R-E Lee JY Park J Jeon K. Association of plasma level of high-mobility group box-1 with necroptosis and sepsis outcomes. Sci Rep (2021) 11:9512. doi: 10.1038/s41598-021-88970-6 33947887 PMC8097071

[98] Ruskowski K Neb H Talbot SR Choorapoikayil S Adam EH von Knethen A. Persistently elevated plasma concentrations of RIPK3, MLKL, HMGB1, and RIPK1 in patients with COVID-19 in the intensive care unit. Am J Respir Cell Mol Biol (2022) 67:405–8. doi: 10.1165/rcmb.2022-0039LE PMC9447136 35385375

[99] Siempos II Ma KC Imamura M Baron RM Fredenburgh LE Huh J-W. RIPK3 mediates pathogenesis of experimental ventilator-induced lung injury. JCI Insight (2018) 3:e97102. doi: 10.1172/jci.insight.97102 29720570 PMC6012515

[100] Dellinger RP Bagshaw SM Antonelli M Foster DM Klein DJ Marshall JC. Effect of targeted polymyxin b hemoperfusion on 28-day mortality in patients with septic shock and elevated endotoxin level: The EUPHRATES randomized clinical trial. JAMA (2018) 320:1455–63. doi: 10.1001/jama.2018.14618 PMC6233793 30304428

