# Corrigendum: RIPK1 in the inflammatory response and sepsis: recent advances, drug discovery and beyond

**DOI:** 10.3389/fimmu.2023.1332633

**Published:** 2023-11-13

**Authors:** Xiaoyu Liu, A-Ling Tang, Jie Chen, Nan Gao, Guoqiang Zhang, Cheng Xiao

**Affiliations:** ^1^ Department of Emergency, China-Japan Friendship Hospital, Beijing, China; ^2^ China-Japan Friendship Hospital, Chinese Academy of Medical Sciences and Peking Union Medical College, Beijing, China; ^3^ Graduate School, Beijing University of Chinese Medicine, Beijing, China

**Keywords:** RIPK1, sepsis, cytokine storm, inflammatory response, PANoptosis

In the published article, an author name was incorrectly written as “Chen Xiao”. The correct spelling is “Cheng Xiao”.

The authors apologize for this error and state that this does not change the scientific conclusions of the article in any way. The original article has been updated.

